# The Lysosomal Calcium Channel TRPML1 Maintains Mitochondrial Fitness in NK Cells through Interorganelle Cross-Talk

**DOI:** 10.4049/jimmunol.2300406

**Published:** 2023-09-22

**Authors:** Dennis Clement, Edina K. Szabo, Silje Zandstra Krokeide, Merete Thune Wiiger, Marianna Vincenti, Daniel Palacios, Young-Tae Chang, Christian Grimm, Sandip Patel, Harald Stenmark, Andreas Brech, Rakesh Kumar Majhi, Karl-Johan Malmberg

**Affiliations:** * Precision Immunotherapy Alliance, Institute for Cancer Research, University of Oslo, Norway; † Department of Cancer Immunology, Institute for Cancer Research, Oslo University Hospital, Oslo, Norway; ‡ Department of Chemistry, Pohang University of Science and Technology, Pohang, Republic of Korea; § Department of Pharmacology and Toxicology, Faculty of Medicine, University of Munich, Munich, Germany; ¶ Department of Cell and Developmental Biology, University College London, London, United Kingdom; ‖ Department of Molecular Cell Biology, Institute for Cancer Research, Oslo University Hospital, Montebello, Oslo, Norway; # Tissue Restoration Lab, Department of Biological Sciences and Bioengineering, Mehta Family Center of Engineering and Medicine, Indian Institute of Technology Kanpur, Kanpur, India; ** Center for Infectious Medicine, Department of Medicine Huddinge, Karolinska Institute, Stockholm, Sweden

## Abstract

Cytotoxic lymphocytes eliminate cancer cells through the release of lytic granules, a specialized form of secretory lysosomes. This compartment is part of the pleomorphic endolysosomal system and is distinguished by its highly dynamic Ca^2+^ signaling machinery. Several transient receptor potential (TRP) calcium channels play essential roles in endolysosomal Ca^2+^ signaling and ensure the proper function of these organelles. In this study, we examined the role of TRPML1 (TRP cation channel, mucolipin subfamily, member 1) in regulating the homeostasis of secretory lysosomes and their cross-talk with mitochondria in human NK cells. We found that genetic deletion of TRPML1, which localizes to lysosomes in NK cells, led to mitochondrial fragmentation with evidence of collapsed mitochondrial cristae. Consequently, TRPML1^−/−^ NK92 (NK92^ML1−/−^) displayed loss of mitochondrial membrane potential, increased reactive oxygen species stress, reduced ATP production, and compromised respiratory capacity. Using sensitive organelle-specific probes, we observed that mitochondria in NK92^ML1−/−^ cells exhibited evidence of Ca^2+^ overload. Moreover, pharmacological activation of the TRPML1 channel in primary NK cells resulted in upregulation of LC3-II, whereas genetic deletion impeded autophagic flux and increased accumulation of dysfunctional mitochondria. Thus, TRPML1 impacts autophagy and clearance of damaged mitochondria. Taken together, these results suggest that an intimate interorganelle communication in NK cells is orchestrated by the lysosomal Ca^2+^ channel TRPML1.

## Key Points

- TRPML1-derived lysosomal calcium regulates mitochondrial organization and fitness.
- Loss of the lysosomal calcium channel TRPML1 leads to mitochondrial dysfunction.
- TRPML1 activity modulates autophagy induction, a prerequisite for mitophagy.

## Introduction

Calcium is a crucial second messenger that orchestrates effector responses, differentiation, and proliferation of cytotoxic lymphocytes (1, 2). The endoplasmic reticulum (ER) is the main Ca^2+^ storage compartment in the cell (2), but mitochondria and lysosomes can also impact global Ca^2+^ homeostasis and act as subcellular Ca^2+^ signaling hubs (3–6). Effective coordination between lysosomes and mitochondria is essential for sensing stress signals and enabling the effective redirection of nutrients (6). These adjustments ensure metabolic adaptation based on nutrient availability and help sustain energy production (7–9). The tumor microenvironment is characterized by low nutrient concentrations, including low glucose, hypoxia, low pH, and high lactate, leading to metabolic stress that impacts tumor-infiltrating lymphocyte function (10–12). Such metabolic restrictions induce mitochondrial fragmentation in tumor-infiltrating NK cells, potentially explaining their reduced cytotoxic function, poor survival, and correlation with negative disease outcomes (11).

Lysosome–mitochondria contact sites play a pivotal role in regulating the morphology of the mitochondrial network (13, 14). These contact sites are adversely affected by genetic defects in *MCOLN1* gene which codes for the lysosomal transient receptor potential (TRP) cation channel, mucolipin subfamily, member 1 (TRPML1), resulting in a severe lysosomal storage disorder mucolipidosis type IV (15). Fibroblasts from mucolipidosis type IV patients have defective lysosome–mitochondrial contacts and dysregulated contact-dependent organellar calcium homeostasis (13). Loss of TRPML1 also leads to elevated mitochondrial reactive oxygen species (ROS) levels and dysfunctional mitochondria (15, 16). TRPML1-mediated lysosomal calcium efflux also plays a crucial role in controlling the expression of essential autophagy genes and, as a result, coordinates the disposal of damaged mitochondria through a process called mitophagy (17–19).

Notably, TRPML1 is widely expressed in immune cells, including NK cells (20). However, rapidly proliferating NK cells exhibit elevated ROS formation and accumulate damaged mitochondria (14). Mitophagy sustains adaptive NK cell survival by eliminating damaged mitochondria and preventing apoptosis (14). In line with this, pharmacological induction of mitophagy through mTOR inhibition or AMPK activation has proven beneficial for NK cell survival (14). This underscores the importance of tight coordination between lysosomes and mitochondria in NK cells. However, little is known about how lysosomal calcium contributes to safeguarding NK cell functionality. Recently, we investigated the molecular correlates behind the superior cytotoxicity of educated NK cells and reported that acute modulation of TRPML1 could enhance the cytotoxic payload of secretory lysosomes in NK cells (20). However, the role of TRPML1 in maintaining mitochondrial fitness and organelle communication in NK cells has not been thoroughly investigated (21, 22). Using a combination of pharmacological and genetic interventions with an imaging-based approach, we demonstrate in the current study that TRPML1-derived Ca^2+^ signals play a role in regulating mitochondrial architecture and function in NK cells. Furthermore, we show that TRPML1, together with AMPK, regulates autophagy in NK cells by modulating LC3 expression. Overall, our data suggest an important role for the lysosomal/mitochondrial Ca^2+^ axis in regulating NK cell fitness.

## Materials and Methods

### Reagents and cell lines

The TRPML1 agonist MK6-83 (Sigma-Aldrich) was dissolved in DMSO and used at 20 μΜ unless specified otherwise. The TRPML1 antagonist ML-SI3 (enamine) was dissolved in DMSO and used at 50 μΜ. The chemical modulators ML-SA1, A-769662, bafilomycin A1, ionomycin, MGR1, and MGR2 were used as indicated in the figure legends, and all were obtained from Sigma-Aldrich. Clinical-grade recombinant human IL-2 (Proleukin) was obtained from Clinigen via Sykehusapoteket (no. 600373). IL-15 was used at 1 or 10 ng/ml (Miltenyi Biotec).

### Cell lines

K562 cells were acquired from American Type Culture collection and cultured in RPMI 1640, supplemented with 10% FCS and penicillin-streptomycin. The NK92 cell line was purchased from German Collection of Microorganisms and Cell Cultures and were cultured in α-MEM (Life Technologies), supplemented with 200 U/ml IL-2, 12.5% FBS, 12.5% horse serum, 2 μΜ l-glutamine, and penicillin-streptomycin (all Life Technologies).

### Gene editing and cultivation of NK-92 cells

Cas9 ribonucleoprotein complexes were assembled by mixing 20 pmol of Cas9 (Synthego) to 180 pmol of TRPML1-specific synthetic guide RNA (5′-ACACGTCAGGCAACGCC-3′, 5′-CTCGGTGGTAGTACCGC-3′, 5′-TCACCAGTAACCACCAT-3′; CRISPRevolution single-guide RNA EZ kit v2, Synthego). A total of 1.5 × 10^5^ NK-92 cells in 5 mM KCl, 15 mM MgCl, 15 mM HEPES, 150 mM Na_2_HPO_4_/NaH_2_PO_4_ (pH 7.7), and 50 mM mannitol were mixed with Cas9 ribonucleoprotein and electroporated using the Lonza 4D-Nucleofector system with pulse code CM-137 (23). For clonal expansion of TRPML1^−/−^ NK92 (NK92^ML1−/−^), we prepared the feeder cells as follows: freshly isolated PBMCs from four healthy donors were pooled and subjected to 100 Gy. Feeder cells were plated with NK-92 media in 96-well U-bottom plates with a density of 5 × 10^6^ cells/ml and incubated at 37°C for 24 h. Single CRISPR-edited NK-92 cells were seeded onto feeders using a FACSAria II cell sorter. Media were replaced weekly until visible cultures were formed. TRPML1-depleted clones were verified using DNA sequencing.

### Generation of viral vectors

The lentiviral expression construct pScalps_TRPML1-mCherry-HA and pScalps_CEPIA2mt was generated by molecular cloning using pCMV-TRPML1-mCherry-HA (gift from Shmuel Muallem) or pCMV CEPIA2mt (gift from Masamitsu Iino, Addgene plasmid no. 58218) as a PCR template. The insert was ligated into BamHI/Not restriction site of pScalps_Puro (gift from Silvia Monticelli, Addgene plasmid no. 99636). Pseudotyped lentiviral particles were produced by cotransfecting LentiX 293T cells (Takara Bio) with pScalps_TRPML1-mCherry-HA or pScalps_CEPIA2mt, packaging vectors pRSV-Rev and pMDLg/pRRE (gifts from Didier Trono, Addgene plasmid nos. 12253 and 12251), and pCMV-VSV-G (gift from Bob Weinberg, Addgene plasmid no. 8454) using Lipofectamine 3000 (Invitrogen). Virus-containing media were harvested and filtrated 48 h after transfection. The titer was determined using LentiX GoStix (Takara Bio). Virus was concentrated using LentiX concentrator (Takara Bio) and stored at −80°C until use.

### Viral transduction of NK-92 cells

For successful transduction of NK-92 cells, we used the following procedure: 2.5 × 10^5^ cells were mixed with a 1.5 multiplicity of infection of lentivirus in a total volume of 250 µl of cell media with 8 µg/ml protamine sulfate and 6 µM Bx795 in a 48-well plate. The plate was centrifuged for 1 h at 900 × *g* at 32°C followed by incubation for 4 h at 37°C. Cells were washed in media and resolved in 0.5 ml of fresh media with a 1.5 multiplicity of infection of the same lentivirus, without addition of transducing agents. Cells were incubated overnight at 37°C. The same procedure was repeated on 2 constitutive days, for a total of six transductions. Flow cytometry was used to evaluate transduction efficiency, and FACS sorting was employed to obtain pure populations. Sorted populations were maintained under 2 μg/ml puromycin selection whenever needed.

### Live cell cytotoxicity and proliferation assays

Ninety-six–well flat-bottom plates (Corning) were coated with 0.01% poly-l-ornithine solution (Merck) for 16 h at 4°C. For the cytotoxicity assay, K562 cells (American Type Culture Collection) cells transfected with IncuCyte NucLight Green lentivirus reagent (Sartorius) for stable expression of nuclear GFP were seeded at a density of 5 × 10^6^ cells/well in a coated 96-well plate. Effector cells were added in the corresponding ratio of 5:1 or 3:1. Control wells contained targets only. The assay plates were scanned every 2 h for a total of 48 h in an IncuCyte S3 (Sartorius) live-cell analysis system, placed in a conventional incubator at 37°C and 5% CO_2_. The area of green fluorescent target cells was quantified over time, using the corresponding IncuCyte S3 software (Sartorius).

### Confocal microscopy staining and analysis

NK92 cells were stained by incubating in a 37°C incubator for 20 min with 300 nM MitoTracker Deep Red (Invitrogen) or 500 nM LysoTracker Red DND (Invitrogen) for 30 min in complete α-MEM media. Cells were fixed with 4% paraformaldehyde, washed with PBS, and counterstained with 5 μg/ml Hoechst for 5 min. To show interaction with lysosomes, as well as colocalization with a lysosome or F-actin network, cells were permeabilized with PBS containing 0.1% saponin and 1% BSA and stained with Alexa Fluor 488–tagged anti-LAMP1 Ab (clone H4A3, BioLegend), granzyme B Ab (clone GB11, BD Biosciences) in room temperature for 4 h, or phalloidin (Thermo Fisher Scientific) for 1 h. Confocal imaging was performed at ×8 zoom using a ×63 oil immersion objective on a LSM 880 microscope (Zeiss). Super-resolution microscopy images of single cells were acquired in the Airyscan mode. Images were processed using Zen Blue (Zeiss), Fiji, and Imaris (Oxford Instruments) software.

### Electron microscopy and analysis

For representative images of mitochondria and quantification of mitochondrial morphology, pellets of NK92 cells were fixed in 2% glutaraldehyde and 4% paraformaldehyde in 0.1 M PHEM (60 mM PIPES, 25 mM HEPES, 10 mM EGTA, and 2 mM MgCl_2_ at pH 6.9) buffer overnight, and subsequently embedded in 2% agarose at 60°C before postfixation in 2% osmium (Electron Microscopy Sciences) and 1% ferricyanide in 0.1 M PHEM buffer on ice for 1 h. Pellets were washed with water to remove osmium residues, after which cell pellets were dehydrated in a graded ethanol series, embedded in EPON, and polymerized for 48 h. Ultrathin sections of 100 nm were cut using an Ultracut UCT ultramicrotome (Leica) and transferred onto 100-mesh carbon-coated grids for imaging in the transmission electron microscope.

For quantification of mitochondrial intercristae distances, cell pellets were fixed in 0.1% glutaraldehyde and 4% paraformaldehyde in 0.1 M PHEM buffer overnight, and subsequently embedded in 10% gelatin, infiltrated with 2.3 M sucrose, and frozen in liquid nitrogen. Samples were stored in liquid nitrogen until sectioning. Ultrathin sections of 70–80 nm were cut with a Leica Ultracut (equipped with a UFC cryochamber) at −110°C, then picked up with a 50:50 mixture of 2.3 M sucrose and 2% methyl cellulose and placed onto 100-mesh carbon-coated grids. Samples were contrasted with uranyl acetate before imaging. Microscopy was done using JEOL JEM-1230 at 80 kV and images acquired using ITEM software with a Morada camera (Olympus). All quantifications were done using ImageJ.

### Mitochondrial stress assay

NK cells were treated as indicated in the figure legends or left untreated. The cells were incubated with MitoTracker Green, MitoProbe TMRM (Thermo Fisher Scientific), or ATP-Red, Mito Thermo Yellow, and the control probes GTP green and ER Thermo Yellow (all provided by Young-Tae Chang, Pohang University of Science and Technology) for 10–30 min at 37°C. Subsequently, samples were washed twice in warm cell culture medium, resuspended in PBS with 2% FCS and immediately acquired on an LSR II flow cytometer (BD Biosciences). For mitochondrial ROS detection, cells were loaded with MitoSOX Red (Thermo Fisher Scientific) and incubated for 8 min at 37°C. The MitoSOX signal was detected on an LSM 880 confocal laser scanning microscope (Zeiss) and quantified using the software Zen Blue (Zeiss) and Fiji.

### Proximity ligation assay

NK cells were stained with mouse anti–voltage-dependent anion-selective channel (VDAC1) (ab14734, Abcam) and rabbit anti-Rab7 (D95F2, Cell Signaling Technology) Abs to visualize mitochondria–lysosome contact sites. The Duolink proximity ligation assay (PLA) kit (Sigma-Aldrich) was used, in conjunction with the Duolink PLA probe anti-mouse MINUS and anti-rabbit PLUS, and the Duolink detection reagent FarRed (Sigma-Aldrich) following the manufacturer’s recommendations. Fluorescent PLA signal was detected via confocal microscopy. The PLA signal was quantified as spot count per cell (nucleus) using Fiji software.

### Calcium flux assay

The genetically encoded, mitochondria-targeted pScalps_CEPIA2mt calcium reporter was transduced into NK92 cells (American Type Culture Collection, CRL-2407), sorted by flow cytometry, and used to measure mitochondrial calcium flux via flow cytometry. To measure the cytosolic calcium levels, these NK92 cells were also loaded with 2 µM Indo-1-AM (Thermo Fisher Scientific) in the presence of Pluronic F-127 (Sigma-Aldrich) and 2.5 mM probenecid (Thermo Fisher Scientific) in HBSS (Life Technologies) with 2 mM calcium and 2% FCS for 30 min at 37°C. Subsequently, full α-MEM was added and incubated for another 30 min at ambient temperature to enable intracellular trapping of Indo-1. The loaded cells were surface stained with CD45-BV785 (BioLegend) and LIVE/DEAD near-infrared marker (Thermo Fisher Scientific) and washed once. Samples were kept on ice and warmed in a 37°C water bath for 3 min prior to acquisition. Data were acquired on a FACSymphony A5 (BD Biosciences) equipped with a UV laser. Calcium flux was triggered by adding MK6-83 or bafilomycin A1 as indicated in the graphs. Ionomycin (1 µM) was added at the end of the assay to show maximum responsiveness of the system. CEPIA_mt_ fluorescence and the ratiometric Indo-1 fluorescence were collected in the FACSDiva flow cytometer (BD Biosciences), and the FCS files were exported to FlowJo (BD Biosciences) for further analysis.

### Immunoblotting and Abs

Cells were harvested by centrifugation, washed in ice-cold PBS, and the pellets were snap-frozen in liquid nitrogen. Frozen cell pellets were lysed in Pierce RIPA lysis buffer (Thermo Fisher Scientific), supplemented with 1× protease and phosphatase inhibitor mix (Invitrogen, Thermo Fisher Scientific), and incubated on ice for 30 min. To further disrupt the cells, the solution was vortexed and homogenized with hydrodynamic shearing using a 22G needle 5–10 times. After 30 min on ice, the solution was centrifuged at 10,000 × g and the resulting supernatant was the total protein lysate. The total protein content in the lysate was measured in a Pierce bicinchoninic acid (BCA) assay with BSA as the standard (Thermo Fisher Scientific), and an equal protein amount of each lysate was applied to the gel under reducing conditions. The proteins were transferred to a polyvinylidene difluoride membrane (Life Technologies) with an iBlot2 dry blotter, and the membrane was blocked according to the Ab manufacturers’ recommendations and incubated overnight with primary Ab at 4°C. The following Abs were used: β-actin (Sigma-Aldrich), Drp1 (clone D6C7), Drp1^pS616^ (no. 3455), Mfn1 (clone D6E2S), Mfn2 (clone D1E9), Opa1 (clone D6U6N), and LC3A/B (no. 4108) were all purchased from Cell Signaling Technologies. VDAC1 (ab14734) and mitochondrial calcium uptake 1 (MICU1) (ab190114) were from Abcam. After washing and incubation with a secondary HRP-conjugated Ab (Cell Signaling Technologies) for 1 h at room temperature, the Ag signal was detected upon incubation with a suitable Pierce ECL substrate (Thermo Fisher Scientific) and an iBright CL1500 imager. Protein bands were quantified with the iBright analysis software (Thermo Fisher Scientific) and normalized to a loading control.

### Metabolic assays

Metabolic flux analysis was performed on a Seahorse XF96 metabolic flux analyzer (Agilent Technologies). Briefly, 96-well flat-bottom plates (Agilent Technologies) were coated with 15 µg/ml Cell-Tak (Corning) overnight at 4°C. NK92 cells (3 × 10^5^ cells/well) were resuspended in Seahorse XF RPMI medium (Agilent Technologies), supplemented with 2 mM l-glutamine, 2 mM pyruvate, and 10 mM d-glucose (Agilent Technologies) and spun down at 200 × *g* for 2 min. The cell-loaded plates were incubated for 1 h in 0% CO_2_ atmosphere at 37°C. Subsequently, the cartridge was loaded with oligomycin A, CCCP, and rotenone/antimycin A1 (Sigma-Aldrich) in DMSO. The protocol for a mitochondria stress test was selected in the Seahorse XF software (Agilent Technologies). The data were exported to Excel and plotted with GraphPad Prism 9.

For the detection of glucose and lactate in cell culture supernatants, a Glucose-Glo (Promega) and a Lactose-Glo luciferase assay (Promega) were used according to the manufacturer’s instructions. NK92 cells were cultured in a poly-l-ornithine–coated 96-well flat-bottom plate (Corning). Normal NK92 α-MEM supplemented with 200 U/ml IL-2, 12.5% FBS, 12.5% horse serum, l-glutamine, and penicillin-streptomycin was used. Cells were cultured for 96 h in an Incucyte S3 (Sartorius) live cell analysis system. At the end of the period, phase-contrast data were used to normalize for the cell number in each well. Cell culture supernatants were collected and diluted 1:200 in PBS and stored at −20°C until acquisition on a 96-well EnVision 2105 multimode plate reader (PerkinElmer).

### Autophagy assays

Autophagy levels were monitored via measurement of the lipidated LC3-II form by flow cytometry, according to the manufacturer’s instructions, using the Guava autophagy detection kit (Luminex). Briefly, NK cells were selectively permeabilized to extract the cytosolic from of LC3 while protecting the membrane-bound LC3-II form. In a subsequent step, LC3 was stained with an anti-LC3 FITC Ab (clone 4E12, Guava autophagy detection kit, Luminex). For the measurement of autophagic flux, the CYTO-ID autophagy detection kit 2.0 was used following the manufacturer’s instructions (Enzo Life Sciences). Autophagy assays were acquired on an LSR II flow cytometer (BD Biosciences).

### RNA sequencing and RT-PCR analysis

Total RNA extraction was performed, as previously described (24), using 500 ng of RNA transcribed to cDNA with a SuperScript cDNA synthesis kit (Life Technologies) according to the manufacturer’s instructions. Gene expression was analyzed by a SYBR Green–based assay (Life Technologies) in a Mic quantitative real-time PCR cycler (Bio Molecular Systems Biology) using gene-specific RT^2^ Profiler PCR primers (Qiagen). RNA sequencing (RNA-seq) was performed using single-cell tagged reverse transcription, a highly multiplexed method for single-cell RNA-seq (25). The detailed procedure is available in our online resource (https://rnaseq.malmberglab.com/) (26).

### Statistical analysis

Graphs represent data from three independent experiments as mean ± SEM, unless stated otherwise. Graphs were generated and statistics were calculated with the help of Prism 9 (GraphPad Software). For the comparison of two groups, an unpaired Student *t* test was applied. For the comparison of more than two groups, an ANOVA test was performed or a Brown-Forsythe and Welch ANOVA test. Statistical significance is indicated as follows: *p < 0.05, **p < 0.01, ***p < 0.001, and ****p < 0.0001 (ns, not significant).

## Results

### Disruption of TRPML1 leads to calcium overload and mitochondrial stress in NK cells

**FIGURE 1.**
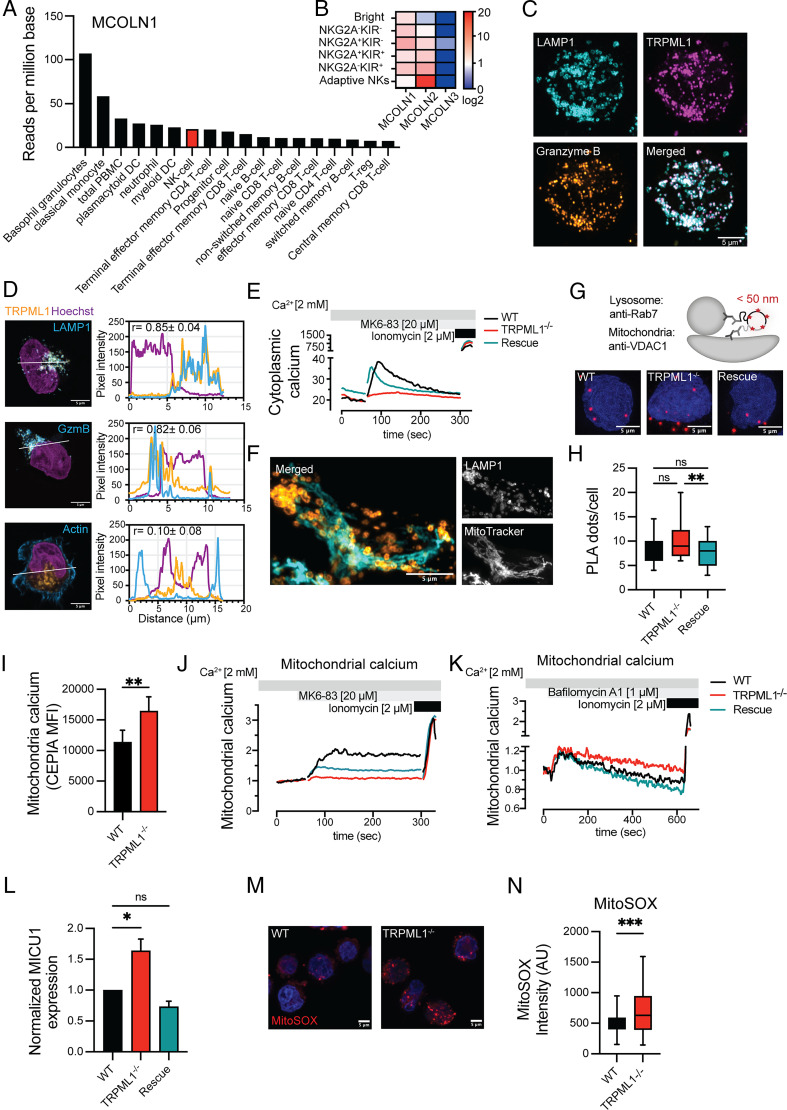
Disruption of TRPML1 leads to calcium overload and mitochondrial stress. (**A**) Normalized transcripts per million expression values of *MCOLN1* gene expression across immune cells isolated from total PBMCs of healthy donors (from Human Protein Atlas) (68, 69). (**B**) Heatmap comparing TRPML channels (*MCOLN1–3*) mRNA expression levels from PBMCs of healthy blood donors (*n* = 10). Indicated NK cell subsets were sorted according to their maturation levels and sequenced using single-cell–tagged reverse transcription (https://rnaseq.malmberglab.com/) (26). (**C**) Subcellular localization of mCherry-tagged TRPML1 in NK92^WT^ cells. Lysosomes were stained with anti-LAMP1 and anti-granzyme B Abs. White appearance reflects maximum overlap between the three proteins. Scale bar, 5 μm. (**D**) Representative deconvolved images with line profiles depicting the extent of colocalization of mCherry-TRPML1 (orange) with lysosomal markers LAMP1 and granzyme B or peripheral F-actin (cyan) in NK92^WT^ cells quantified from 8 to 10 cells each from three independent sets (*n* = 24–30). Median value of Pearson coefficients with SD is displayed. Hoechst staining (purple) depicts the nucleus. Scale bars, 5 μm. (**E**) Representative line graph depicting calcium mobilization in NK92^WT^, NK92^ML1−/−^, and NK92^rescue^ cells upon treatment with the TRPML1 agonist MK6-83 (20 µM), as measured by Indo-1 intensity via flow cytometry. Ionomycin was added at the last 30 s to quantify the maximal Ca^2+^ flux. (**F**) AiryScan super-resolution imaging of NK92^WT^ cells stained with mitochondrial marker MitoTracker Orange and anti-LAMP1 (lysosomal marker) depict physical association of most lysosomes with mitochondria. Scale bar, 5 μm. (**G**) Model and representative images of NK92^WT^, NK92^ML1−/−^, and NK92^rescue^ cells depicting proximity ligation assay (PLA) to visualize the mitochondria–lysosome membrane contact sites (red) between lysosomes (Rab7) and mitochondria (VDAC1). DAPI (blue) served as a nuclear counterstain. Scale bars, 5 μm. (**H**) Quantitative analysis of PLA signals from the indicated cell lines analyzed from 38 to 47 cells of each cell line from two independent experiments. Whiskers show 5th to 95th percentile. Bars show the median values of PLA signals. (**I**) Flow cytometry–based quantification of the mitochondrial calcium sensor CEPIA_mt_ fluorescence intensity in NK92^WT^ and NK92^TRPML1−/−^ cells at resting stage; *n* = 7 independent experiments. (**J**) Mitochondrial calcium flux in NK92 cells expressing the mitochondrial targeted calcium sensor CEPIA_mt_, triggered by the TRPML1-agonist MK6-83 (20 μM). Ionomycin (2 μM) was added as positive control during the last 30 s of recording to show maximum calcium response. Baseline was recorded for 60 s. Data are shown as fold change of fluorescence intensity at indicated time points normalized to corresponding intensity at the initial time point (*F_x_*/*F*_0_). (**K**) Lysosomal calcium release was triggered by the TRPML1-independent vacuolar-type ATPase inhibitor bafilomycin A1 (1 μM) in NK92 cells. Mitochondrial calcium flux was recorded as in (J). Ionomycin acts as positive control at the end of the recording. (**L**) Western blot analysis of mitochondrial calcium uniporter 1 (MICU1) expression in NK92^WT^, NK92^TRPML1−/−^, and NK92^rescue^ cells, normalized to β-actin levels. *n* = 3. (**M**) Representative confocal microscopy images of live cell imaging depicting NK92 cells stained with a mitochondrial ROS indicator (MitoSOX). DAPI (blue) was used as a nuclear counterstain. Scale bars, 5 μm. (**N**) Quantification of MitoSOX pixel intensity values (in arbitrary units [AU]) depicting mitochondrial ROS levels in NK92 cells, from 14 to 16 cells of each condition; *n* = 3 independent experiments. Data are presented as mean ± SEM. One-way ANOVA followed by a Welch multiple comparison test was performed in (H). An ordinary one-way ANOVA multi comparison test was performed in (K). A paired *t* test (J) and an unpaired *t* test with a Welch correction was used to compare the groups in graph (N). *p < 0.05, **p < 0.01, ***p < 0.001. ns, not significant.

Analysis of the Human Protein Atlas database revealed that the lysosome-specific Ca^2+^ channel, TRPML1, encoded by the *MCOLN1* gene, is highly expressed in myeloid cells, closely followed by NK cells (Fig. 1A). Total RNA-seq analysis of NK cell subsets revealed consistent abundance of *MCOLN1* transcripts across all maturation states (Fig. 1B). In contrast, *MCOLN2* expression was highest in adaptive NK cells, whereas *MCOLN3* expression was undetectable (Fig. 1B). In the resting human NK cell line NK92, mCherry-tagged TRPML1 resided in cytotoxic granules, as illustrated by its colocalization with the lysosomal marker LAMP1 (*r*^2^ = 0.85 ± 0.04) and granzyme B (*r*^2^ = 0.82 ± 0.06) (Fig. 1C, 1D). No TRPML1 was detected beyond the actin cortex (*r*^2^ = 0.10 ± 0.08) or on the plasma membrane, confirming its specific localization in the lysosome under resting conditions (Fig. 1D). We used CRISPR/Cas9 to genetically ablate the *MCOLN1* gene in the human NK cell line NK92, selected homozygous NK92^ML1−/−^ clones, and created a rescue line by overexpressing mCherry-tagged TRPML1. Both the NK92^ML1−/−^ and NK92^rescue^ cells showed viability and cell proliferation rates comparable to the NK92 wild-type (NK92^WT^) cells (Supplemental Fig. 1A). Pharmacological activation by the TRPML1 agonist, MK6-83, triggered an almost immediate cytoplasmic Ca^2+^ elevation in NK92^WT^ and NK92^rescue^ cells, indicative of robust lysosomal Ca^2+^ efflux via TRPML1 (Fig. 1E). In contrast, the NK92^ML1−/−^ cells showed little to no response to agonistic stimulation. All three cell lines demonstrated a robust response to ionomycin, indicating that the rest of their ion channels were intact and functional.

Direct physical interaction between the lysosome and mitochondria in epithelial cells facilitates TRPML1-mediated lysosomal Ca^2+^ efflux to be taken up by the mitochondria (13, 18). Super-resolution imaging with MitoTracker and LAMP1 staining suggested that a considerable fraction of lysosomes was physically in contact with mitochondria in resting NK92 cells (Fig. 1F). This observation was corroborated by PLA using Abs against the mitochondrial ion channel VDAC1 and Rab7, primarily expressed on lysosomes and late endosomes (27) (Fig. 1G). Interestingly, NK92^ML1−/−^ cells tended to have a higher number of lysosome/late endosome–mitochondria contact sites compared with NK92^WT^ and NK92^rescue^ cells (Fig. 1G, 1H).

To unravel the functional implications of augmented interaction between the two organelles, we next investigated the nature of interorganellar calcium transfer that occurs at lysosome–mitochondria membrane contact sites (13) by expressing a mitochondria-targeted Ca^2+^ sensor in NK92 cells. The correct localization of the mitochondrial Ca^2+^ sensor was confirmed by colocalization with MitoTracker staining (Supplemental Fig. 1B). At baseline, NK92^ML1−/−^ cells were characterized by significantly higher mitochondrial Ca^2+^ levels as compared with NK92^WT^ cells (Fig. 1I). Upon normalizing the baseline mitochondrial Ca^2+^ levels, we observed that in NK92^WT^ cells the mitochondria efficiently sequestered lysosomal Ca^2+^ efflux triggered by the TRPML1 agonist MK6-83, indicated by a rise in mitochondrial Ca^2+^ (Fig. 1J). NK92^ML1−/−^ cells did not show a detectable increase in mitochondrial Ca^2+^ levels, whereas the rescue cells showed slightly lower levels of mitochondrial Ca^2+^ sequestration as compared with NK92^WT^ cells. Ionomycin stimulation induced rapid and nearly equal elevation in mitochondrial Ca^2+^ levels in all cell lines. Importantly, mitochondrial Ca^2+^ reached sustained elevated levels following stimulation with bafilomycin A1, an established inducer of TRPML1-independent lysosomal Ca^2+^ release, indicating that the Ca^2+^-buffering capacity of NK92^ML1−/−^ cells remained intact (Fig. 1K).

Interestingly, NK92^ML1−/−^ cells also had a significantly higher expression of MICU1 protein, which acts as a gatekeeper of the mitochondrial Ca^2+^ uniporter channel complex (Fig. 1L, Supplemental Fig. 1C). Recently, MICU1 has been shown to regulate mitochondrial Ca^2+^ levels and mitochondrial cristae independent of the mitochondrial Ca^2+^ uniporter channel complex (28). Notably, the expression levels of the major mitochondrial calcium-conducting channel VDAC1 remained unaltered in all the three cell types (Supplemental Fig. 1C). Mitochondrial Ca^2+^ overload can stress mitochondria and is one cause of excessive ROS production (29, 30). In agreement with this notion, NK92^ML1−/−^ NK cells showed higher levels of mitochondrial ROS, indicating the stressed state of mitochondria (Fig. 1M, 1N).

### Disruption of TRPML1 leads to mitochondrial fragmentation in NK cells

**FIGURE 2.**
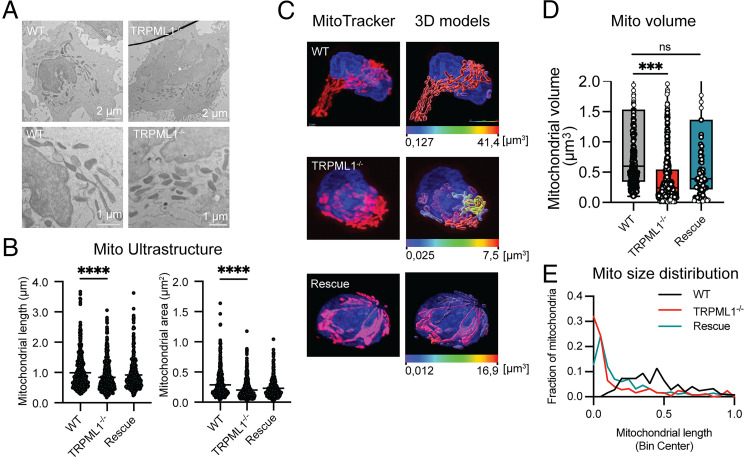
CRISPR-mediated deletion of TRPML1 leads to mitochondrial fragmentation. (**A**) Representative electron micrographs depicting mitochondrial morphology of NK92^WT^, NK92^ML1−/−^, and NK92^rescue^ cells. Scale bars, 2 μm for whole-cell images (top row) and 1 μm for magnified images (bottom row). (**B**) Quantification of mitochondrial length and area by the image analysis software Fiji; *n* = 319–544 cells. (**C**) Representative AiryScan super-resolution microscopy images of MitoTracker-stained NK92 cells depicting detailed mitochondrial morphology. Scale bar, 2 μm. Three-dimensional remodeling and volume rendering of the mitochondrial network was performed with the help of Imaris software (Bitplane). Mitochondrial volume statistics (μm^3^) are color encoded. (**D**) Quantitative analysis of the segmented mitochondrial volume (μm^3^) as determined by Imaris software from high-magnification, super-resolution single-cell images. Compiled data from at least 20 cells per condition from two independent experiments are shown. (**E**) Histogram showing the mitochondrial volume distribution across the dataset. Binned data are shown with relative frequency distribution. Compiled data from at least 20 cells per condition from two independent experiments are shown. Statistical analyses were performed using a Kruskal–Wallis test with a Dunn multiple comparison test (B) or one-way ANOVA for multiple comparisons (D). ***p < 0.001, ****p < 0.0001. ns, not significant.

Mitochondrial oxidative stress has been linked to mitochondrial fragmentation as a mechanism to relieve cellular stress (31). Analysis of the two-dimensional ultrastructure of mitochondria by transmission electron microscopy demonstrated a significant extent of mitochondrial fragmentation induced by disruption of TRPML1, compared with NK92^WT^ and NK92^rescue^ cells, evident from their lower mitochondrial length and area (Fig. 2A, 2B). Furthermore, three-dimensional super-resolution microscopy of MitoTracker-stained mitochondria followed by surface rendering of the images revealed the presence of elongated and highly connected mitochondria in NK92^WT^ cells, whereas the NK92^ML1−/−^ cells showed highly fragmented mitochondria with reduced mitochondrial volume (Fig. 2C–E). Confocal microscopy of fixed cells as well as live cells showed that NK92^WT^ cells were characterized by large interconnected tubular mitochondrial networks, which constituted >50% of the total mitochondrial volume, whereas the NK92^ML1−/−^ cells had predominantly fragmented, spherical mitochondria (Fig. 2E, Supplemental Fig. 2A). Reintroduction of TRPML1 rescued the phenotype (Fig. 2B–E). Western blot analysis indicated no apparent changes in the expression of any of the mitochondrial fission–fusion markers Mfn1, Mfn2, Opa1, and phospho-Drp1^pS616^, despite an increase in total Drp1 levels, suggesting that the restructuring of the mitochondrial network following *MCOLN1* deletion was Mfn2-, Opa1-, and phospho-Drp1–independent (Supplemental Figs. 1C, 2B). In addition, phospho-Drp1 also remained unaltered upon activation or inhibition of TRPML1 in primary NK cells (Supplemental Fig. 2C). In summary, the lysosomal TRPML1 channel plays a crucial role in maintaining mitochondrial homeostasis in NK cells.

### TRPML1 deficiency affects mitochondrial fitness and ultrastructure

**FIGURE 3.**
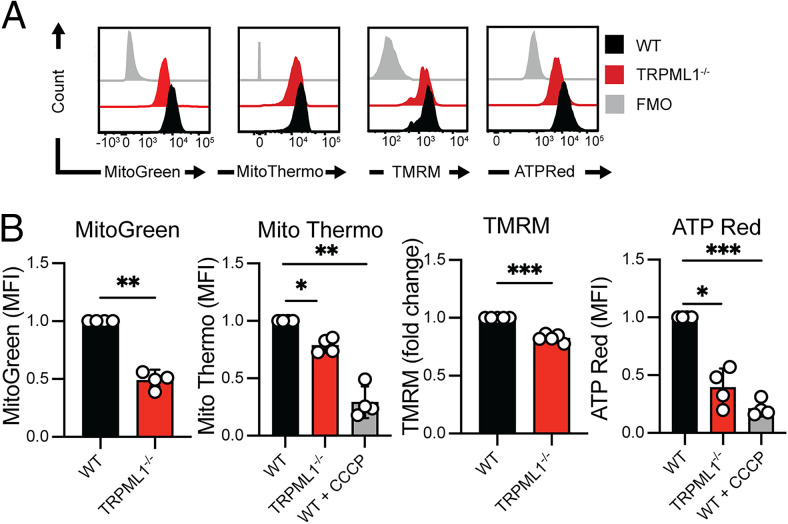
TRPML1 deficiency affects mitochondrial fitness. (**A** and **B**) Fluorescence intensity histograms (A) and corresponding bar graphs (B) depicting fluorescence intensities of the mitochondrial mass–sensitive MitoTracker Green, mitochondrial temperature probe MitoThermo, mitochondrial membrane potential–sensitive MitoProbe TMRM, and mitochondrial ATP-sensitive dye ATP-Red in NK92^WT^ (black), NK92^TRPML1−/−^ (red), and fluorescence minus one (FMO, gray) control are shown. The mitochondrial decoupler CCCP (1 μM) was used as a negative control for ATP-Red and as a positive control for MitoThermo, whose fluorescence intensity correlates inversely with mitochondrial temperature; *n* = 3–4 independent experiments. Unpaired *t* tests were used to compare between NK92^WT^ and NK92^ML1−/−^ cells, and a one-way ANOVA was used to statistically compare the groups. *p < 0.05, **p < 0.01, ***p < 0.001.

To assess the consequences of mitochondrial fragmentation in NK92^ML1−/−^ cells, we performed flow cytometric evaluation of cells using membrane-permeable mitochondrial probes. NK92^ML1−/−^ cells exhibited a 50% reduction in MitoTracker Green intensity, indicating a significant decrease in mitochondrial mass (Fig. 3A, 3B). Using Mito Thermo Yellow, whose mean fluorescence intensity is inversely correlated with mitochondrial temperature, we discovered that NK92^ML1−/−^ cells have a higher mitochondrial temperature (Fig. 3A, 3B). An increase in mitochondrial temperature indicates heightened mitochondrial stress and decoupling of the electron transport chain (32). Indeed, NK92^ML1−/−^ cells showed lower mitochondrial membrane potential than did their NK92^WT^ counterpart (Fig. 3A, 3B). Furthermore, disruption of mitochondrial membrane potential led to lower mitochondrial ATP levels in NK92^ML1−/−^ cells detected by the mitochondrial ATP-selective reporter ATP-Red (Fig. 3A, 3B). As a control, NK92^WT^ cells were treated with the mitochondrial uncoupling agent CCCP, which mimicked mitochondrial dysfunctions as observed in NK92^ML1−/−^ cells (Fig. 3A, 3B).

**FIGURE 4.**
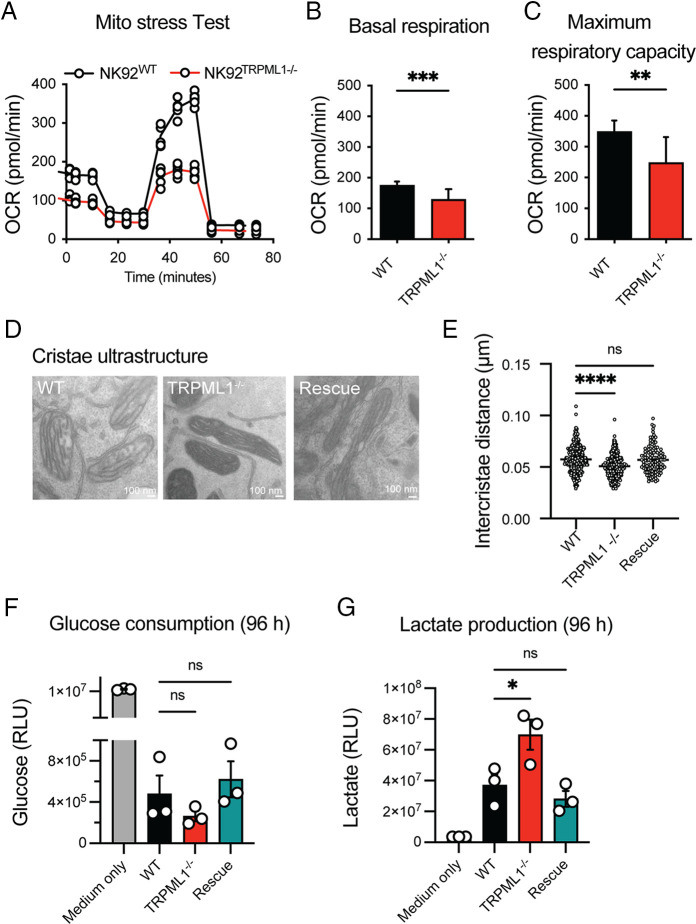
Ultrastructural changes in mitochondria impact cellular metabolism of NK cells. (**A**) Oxygen consumption rate (OCR) during the mitochondrial stress test, depicting the basal respiration and maximal respiratory capacity of NK92^WT^ and NK92^ML1−/−^ cells. A pooled analysis of two independent experiments performed as three replicates in each experiment is shown. (**B** and **C**) Quantification of basal respiration rate and maximum respiratory capacity of NK92^WT^ and NK92^ML1−/−^ cells observed from their oxygen consumption rates as described in (A). (**D**) Representative electron micrographs of mitochondrial ultrastructure from NK92^WT^ and NK92^ML1−/−^, NK92^rescue^ cells. Scale bars, 100 nm. (**E**) Quantification of the mitochondrial cristae density in resting NK92 cells as depicted in (D). Pooled analysis of 117–442 mitochondria from 18–22 cells from two independent experiments were analyzed. (**F** and **G**) Glucose consumption and lactate production of respective NK92 cell lines measured in the cell culture supernatants after 96 h of cell growth. Bioluminescence values (relative light unites [RLU]) corresponding to the glucose (D) or lactate concentrations (E) are shown in the graphs. Data were derived from three independent experiments. Cell culture medium without cells was used as the baseline in the assay. Statistical analysis was performed using a Welch *t* test (B and C) and Kruskal–Wallis test with a Dunn multiple comparison test (E) and one-way ANOVA to compare the groups (F and G). *p < 0.05, **p < 0.01, ***p < 0.001, ****p < 0.0001. ns, not significant.

Next, we explored how the fragmented mitochondria in NK92^ML1−/−^ cells affected mitochondrial bioenergetics and its relationship to the ultrastructural architecture. Measurement of the oxygen consumption rate revealed a lower basal respiration rate and lower maximal respiration rate in NK92^ML1−/−^ cells (Fig. 4A–C). Mitochondria produce most of their ATP through oxidative phosphorylation carried out by the electron transport chain proteins located in the highly invaginated inner mitochondrial membrane, termed cristae. Therefore, cristae organization impacts oxidative phosphorylation and cellular metabolism (33). Ultrastructural analysis using transmission electron microscopy images revealed collapsed mitochondrial cristae in NK92^ML1−/−^ cells, evident from reduction in the intercristae distance (Fig. 4D, 4E). The cristae arrangement could be partially restored in NK92^rescue^ cells. Opa1 levels were slightly lower in NK92^ML1−/−^ NK cells and restored in rescue cells (Supplemental Fig. 2B). Collapsed cristae, together with reduced Opa1 levels, cannot support optimal accumulation of mitochondrial ATP synthase in the cristae to maintain adequate oxidative phosphorylation (34), forcing the cells to switch to nonmitochondrial modes of energy production, such as glycolysis (35). Indeed, NK92^ML1−/−^ cells consumed more glucose from the media in 96-h cultures, showing a higher glycolytic rate compared with NK92^WT^ and NK92^rescue^ cells (Fig. 4F). This was concomitant with higher lactate production in NK92^ML1−/−^ cells, due to higher anaerobic glycolysis (Fig. 4G). Reintroduction of TRPML1 normalized lactate production to baseline WT levels. RT-PCR analysis of key genes involved in mitochondrial respiration revealed no evident changes in NK92^WT^ and NK92^ML1−/−^ cells (Supplemental Fig. 2D). Taken together, these results demonstrate that ultrastructural changes in the mitochondria, including cristae collapse, is accompanied by impaired mitochondrial bioenergetics subjecting the cells to metabolic and energetic stress.

### TRPML1 deficiency impairs autophagy, lysosomal arming, cytokine production, and migration of NK cells

**FIGURE 5.**
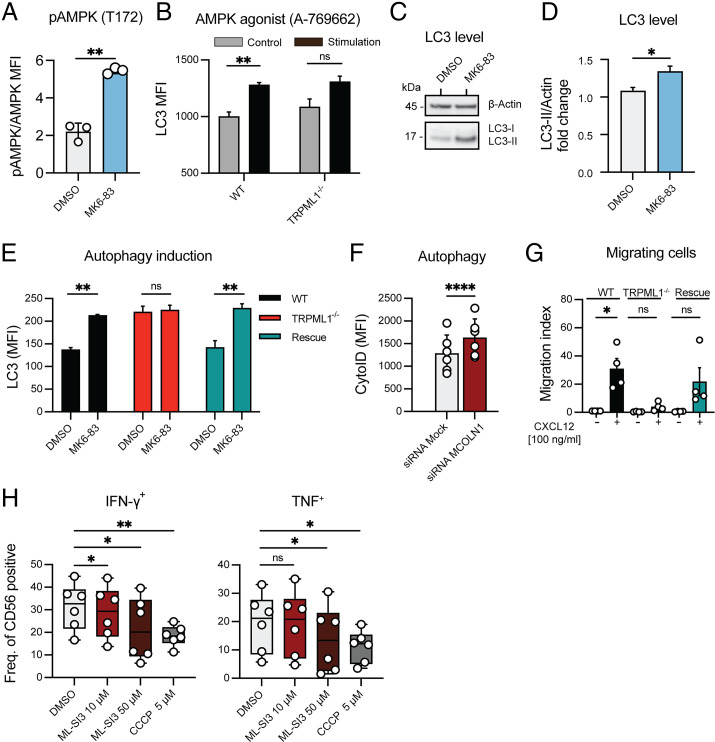
TRPML1 deficiency impairs autophagy, lysosomal arming, and migration of NK cells. (**A**) Relative AMPK phosphorylation of primary NK cells following 5-min stimulation with the TRPML1 agonist MK6-83 (20 μM). Data are shown as the p-AMPK (mean fluorescence intensity [MFI])/AMPK (MFI) ratio. Pooled analysis of is from *n* = 3 donors. (**B**) Autophagy induction measured by flow cytometry–based evaluation of membrane-bound LC3 in NK92 cells following treatment with the AMPK agonist A-769662 (300 μM). Data represent three independent biological replicates. (**C**) Representative immunoblot of the autophagy markers LC3-I and LC3-II in NK92^WT^ cells. Actin was used as a loading control. (**D**) Immunoblot quantification of LC3 levels (fold change) following stimulation with DMSO or MK6-83 (10 μM) for 3 h in NK92 cells. Three independent experiments were analyzed. (**E**) Autophagy induction measured by flow cytometry–based evaluation of membrane-bound LC3 in NK92 cells following treatment with the TRPML1 agonist MK6-83. Data represent three independent biological replicates. (**F**) Comparative analysis of baseline levels of autophagic vacuoles, as determined by flow cytometry of CYTO-ID autophagy detection kit–stained primary NK cells transfected with mock small interfering RNA (siRNA) as control or siRNA targeting *MCOLN1*. Data are shown from six donors. (**G**) Transwell migration assay of NK92 cells. Cells were either left unstimulated or migrated toward a gradient of the chemokine CXCL12 (100 ng/ml) for 1 h. Each dot represents an independent experiment. Data are presented as mean ± SEM of quadruplicates. (**H**) Intracellular cytokine responses of primary NK cells challenged with K562 cells (6-h coculture) in the presence of a TRPML1 antagonist (ML-SI3) in the indicated concentrations. CCCP was used to compromise mitochondrial function. Data are shown from six donors. Paired *t* tests were performed in graphs (A–G) and a one-way ANOVA followed by a Dunnett post hoc test was used for (H). *p < 0.05, **p < 0.01, ****p < 0.0001. ns, not significant.

AMPK is the cellular energy sensor that also regulates lysosomal biogenesis and metabolism. We found that TRPML1 activation by MK6-83 in primary NK cells induced phosphorylation of AMPK (Fig. 5A). AMPK activation induced by cellular stress leads to induction of autophagy, which resolves damaged organelles and cell products (36). Similarly, flow cytometry evaluation revealed that direct activation of AMPK by its specific activator A-769662 (37) induced LC3 accumulation in both NK92^WT^ and NK92^TRPML1−/−^ cells, indicating that TRPML1 acts upstream of AMPK activation and that the downstream AMPK/autophagy pathway remains intact despite TRPML1 depletion (Fig. 5B). Consistent with this direct effect of AMPK activation, pharmacological activation of TRPML1 by its specific activator MK6-83 showed strong induction of autophagy, marked by higher LC3-II levels in NK92^WT^ cells (Fig. 5C, 5D). Flow cytometric quantification of baseline membrane-bound LC3 levels in the NK92 cells confirmed that MK6-83 induced autophagosome accumulation in NK92^WT^ and NK92^rescue^ cells but not in NK92^ML1−/−^ cells (Fig. 5E).

Notably, the NK92^ML1−/−^ cells displayed significantly higher basal level accumulation of autophagosomes compared with their NK92^WT^ counterparts (Fig. 5E). A similar trend of higher autophagosomes was observed in TRPML1-silenced primary NK cells (Fig. 5F). Higher accumulation of autophagosomes could be a consequence of higher mitochondrial ROS, which is known as an important inducer of autophagy in other cells such as macrophages (38). In NK92 cells, autophagosome accumulation was induced by the mitochondria-specific ROS inducer MGR1, but not by its inactive analog MGR2 (Supplemental Fig. 3C, 3D) (39).

Finally, we assessed the impact of pharmacological and genetic modulation of the TRPML1/AMPK axis on NK cell functionality. Lysosomal pH and calcium levels can also be modulated by the metabolic kinase AMPK (40, 41). Moreover, granzyme B and perforin levels were reduced following direct activation of AMPK by A-769662 in primary NK cells (Supplemental Fig. 3A, 3B). NK92^ML1−/−^ cells had no major deficiency in global degranulation responses (Supplemental Fig. 3E) and showed only slightly reduced serial killing capacity (Supplemental Fig. 3F), in line with the fact that this process is less dependent on mitochondrial oxidative phosphorylation (42). However, NK92^ML1−/−^ cells showed reduced migration responses toward a CXCL12 gradient, suggesting that more energy-demanding processes are adversely affected. NK92^WT^ and NK92^rescue^ cells showed robust chemotactic migration (Fig. 5G). Moreover, reduced IFN and TNF production was observed in primary NK cells after treatment with the TRPML1 antagonist, ML-SI3, at 10 and 50 μΜ, followed by K562 tumor cell challenge (Fig. 5H). Thus, collectively our data indicate a close cross-talk between lysosomes and mitochondria of NK cells via TRPML1 channel–mediated regulation of Ca^2+^ dynamics, with specific consequences on NK cell migration and arming of lysosomes with lytic molecules and de novo synthesis of cytokines.

## Discussion

Lysosomal Ca^2+^ signaling can modulate cellular energy homeostasis and regulate mitochondrial dynamics (43). However, limited data are available about these processes in NK cells. These signaling pathways may have potential to make NK cells more stress resilient in the tumor microenvironment. Our results provide insights into how the lysosomal Ca^2+^ channel TRPML1 can orchestrate the remodeling of lysosomes and mitochondria, which is of relevance for NK cell fitness (11, 35).

In recent years the interaction of mitochondria and lysosomes via membrane contact sites has been established (18). During immune responses, adaptations in metabolic flux help to sustain the elevated metabolic needs (7). Mitochondria and lysosomes are highly dynamic and constantly adapt to nutrient availability and receptor input (18, 44). Membrane contact sites facilitate calcium transfer from the lysosome to mitochondria, in a TRPML1-dependent manner (13, 17), illustrating the intimate bidirectional relationship between these organelles. A rise in mitochondrial Ca^2+^ levels is usually tightly coupled to cellular activation and signals energetic demand (45). Other studies have shown by live cell imaging that interorganellar Ca^2+^ transfer depends on tight lysosome–mitochondria membrane tethering (13). Ca^2+^ is released at these membrane–membrane junctions by TRPML1 and sequestered by mitochondria through the outer membrane Ca^2+^ channel VDAC1 (13). Our data identified a direct connection between TRPML1 activity and mitochondrial Ca^2+^ levels, where lysosomes and mitochondria tend to physically interact more, likely to compensate for the absence of quick and robust Ca^2+^ transfer, as seen in TRPML1 knockout cells. We further speculate that the dysregulation of mitochondrial Ca^2+^ levels, at baseline, in TRPML1-deficient cells arises from the increased lysosome–mitochondria tethering, enabling other lysosomal ion channels to deliver Ca^2+^ to mitochondria by virtue of long periods of interaction.

Our results suggest a functional link between lysosomal calcium fluxes and mitochondrial architecture. Likewise, the TRPML1-mediated mitochondrial alterations could prolong the untethering kinetics, which are controlled on the mitochondrial site by TBC1D15 (46). The latter protein controls the untethering kinetics via Rab7 GTP hydrolysis on the lysosomal side (46). However, based on the data presented in the current study, prolonged tethering, alternatively, calcium signaling via other lysosomal ion channels, alterations in lipid and metabolite exchange, or more intimate interaction with the ER cannot be excluded (45, 47, 48). Such mechanisms might force tighter membrane contact sites to reinvigorate the calcium transfer between the organelles. Ultrastructural studies of mitochondria in memory T cells have linked adjustments in metabolic homeostasis to tightening of organelle cross-talk (47). Changes in mitochondrial architecture help memory T cells to gain elevated energetic capacities imperative for their prompt recall responses (49).

In this study we found that genetic ablation of the lysosomal calcium channel TRPML1, in NK92 cells, led to significant deformity in mitochondrial shape, phenotypically detected by mitochondrial fragmentation and cristae condensation. Moreover, TRPML1 deficiency in NK cells led to sustained mitochondrial stress and heightened ROS production and decreased metabolic activity. These findings are in line with reports in cancer cell lines and primary cells from mucolipidosis type IV patients, a disease caused by TRPML1 loss-of-function mutations and characterized by mitochondrial fragmentation (15, 17). Recently, reports in fibroblasts could demonstrate beneficial rewiring of mitochondrial energy capacities by means of mitochondrial remodeling as well as cristae condensation under metabolic stress (50). It is plausible that the cristae remodeling is a consequence of mitochondrial stress and could, at least partially, constitute a compensatory mechanism aiming to promote ATP-linked respiration to sustain cell survival (50). This notion is corroborated by our finding of elevated levels of MICU1 expression levels in TRPML1 knockout cells. Initially, MICU1 has been known to control mitochondrial calcium uptake and thereby safeguard against calcium overload and apoptosis (51). Concomitantly, MICU1 supports the structural integrity of cristae junctions in a calcium-dependent fashion (52), and it fine-tunes mitochondrial membrane potential and proper calcium handling (51). In summary, we have described several mechanisms that may contribute to protect NK cells from calcium challenges and oxidative stress in mitochondria.

How NK cells deal with the consequences of oxidative stress derived from damaged mitochondria remains an open question. Under normal conditions, lysosomes perform a crucial housekeeping role in the maintenance of mitochondria (19, 40, 53). Hence, damaged mitochondria must be degraded to reduce oxidative stress levels (18, 54). Malfunctional mitochondria are dissolved in a self-degrading process, known as mitophagy (54). So far, the role of mitophagy has only been described for the formation of MCMV-dependent memory NK cells (14). During acute MCMV infection, proliferating memory NK cells become apoptotic when they fail to eliminate damaged mitochondria via mitophagy (14). Previous studies have demonstrated how ROS-mediated activation of TRPML1 can initiate autophagy (15, 16, 19, 55). One way involves the calcium-dependent activation of AMPK, downstream of TRPML1 (56). AMPK is one of the master regulators of metabolism and autophagy and is physically situated on the surface of lysosomes and thus very receptive for lysosomal calcium fluxes (43, 56).

The accumulation of damaged and fragmented mitochondria indicates that they were not efficiently resolved in TRPML1-deficient NK92 cells. This could be explained by either dysfunctional TRPML1-dependent initiation of mitophagy or erroneous elimination of autophagosomes (15, 19, 40, 55). At baseline, TRPML1-deficient NK92 cells as well as TRPML-silenced primary NK cells displayed higher levels of membrane-bound LC3-II levels. In parallel, we found accumulation of autophagosomes upon acute pharmacological activation of TRPML1 in NK92^WT^ cells, but not in TRPML1-deficient cells. This indicates that loss of acute TRPML1-derived Ca^2+^ signals may impede downstream activation of autophagy in an AMPK-dependent manner (56). Along the same lines, a role for TRPML1 in autophagic flux via regulation of the lysosomal pH, calcium, and proteolytic activity has been proposed (9, 19, 55–57). Yet, the exact function of TRPML1 in autophagy is still debated (58). Altogether, our data support a model where TRPML1 regulates autophagy in NK cells at different levels; acute TRPML1 stimulation mediates AMPK-dependent induction of autophagy, whereas chronic absence of TRPML1 affects autophagic flux, most likely due to changes in proteolytic activity and membrane dynamics (16, 41, 56, 58–60).

In terms of NK cell effector function, we found slightly increased degranulation in NK92^ML1−/−^ cells, in line with earlier data in primary NK cells (20). However, the modest phenotype in NK92 cells could potentially be explained by the fact that this cell line is IL-2–dependent and characterized by high baseline expression of granzyme B (61). Second, the fusion of lysosomes with the plasma membrane is complex and involves recruitment of a multitude of auxiliary proteins to the surface of the lysosome (62, 63). Based on the recent progress in lysosomal biology, we speculate that TRPML1 could be involved in the extensive remodeling of the lysosomal membrane via microautophagy. Thus, modulation of microautophagy could drastically remodel the lysosomal membrane composition (64–66). Consequently, this would have effects on lysosomal positioning and its fission–fusion dynamics (63). There is evidence that TRPML1-mediated remodeling of cytotoxic granules might extend beyond the luminal part and may as well include the cytoplasmic site of the lysosomal membrane (67).

In conclusion, our work suggests a role for lysosomal calcium signaling in regulating mitochondrial dynamics and initiation of autophagy in NK cells. Further studies are needed to investigate how TRPML1 influences the proteostasis of lysosomal membrane proteins and how mitochondrial adaptation can be modulated to increase the resilience of primary NK cells to metabolic stress in a more physiological setting. This knowledge could potentially improve the performance of cancer-targeted immunotherapy.

## Supplementary Material

Supplemental 1 (PDF)
