# Supplementary material for: The Lysosomal Calcium Channel TRPML1 Maintains Mitochondrial Fitness in NK Cells through Interorganelle Cross-Talk
Source: J Immunol. 2023 Sep 22;211(9):1348–58. doi: 10.4049/jimmunol.2300406 (PMC10579149; doi:10.4049/jimmunol.2300406)
Supplement: Supplemental 1 (PDF) [file JI_2300406_Supplemental_1.pdf]

Figure S1

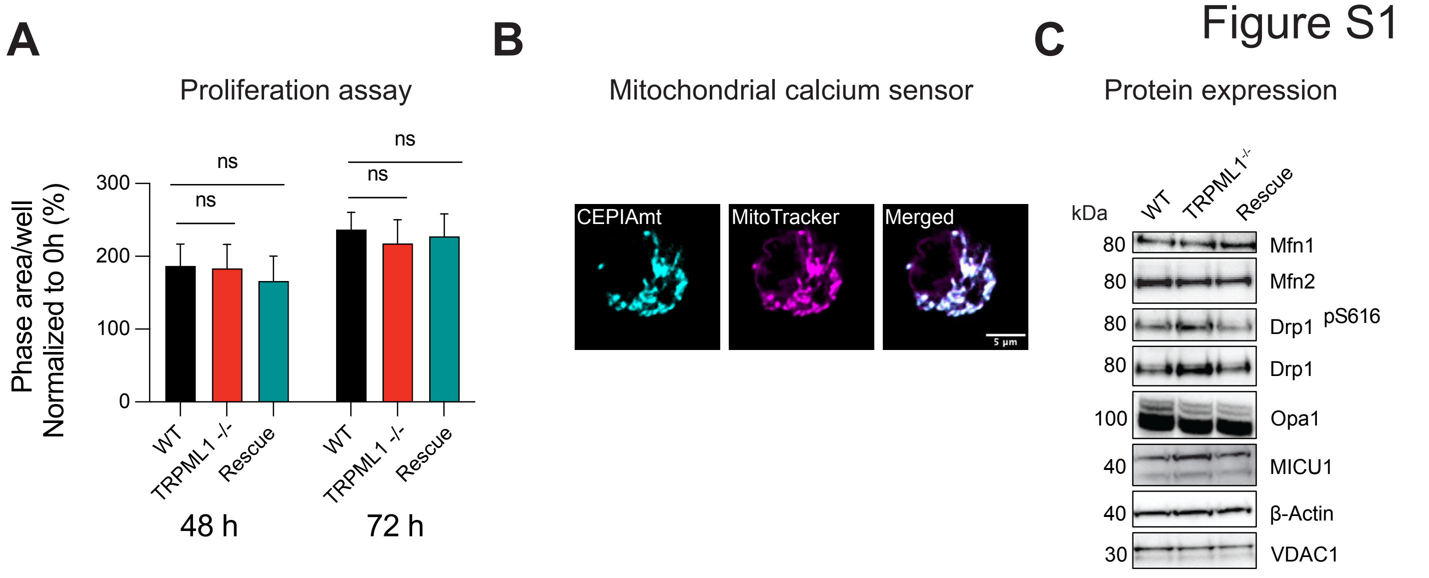

Supplementary Figure 1. (A) Proliferation assay of NK92 cells, seeded in a 96 flat-bottom plate and incubated in an Incucyte S3 live cell imaging system. Quantification of phase contrast images at 48 h and 72 h is plotted to change of confluency in percentage (cell area/well), normalized to time point 0h. Pooled analysis of n=5 independent experiments. (B) Representative confocal microscopy images of NK92WT and NK92TRPML1-/- cells stably expressing the mitochondrial-targeted calcium sensor CEPIAmt depicting co-localization with MitoTracker staining. (C) Representative immunoblot depicting indicated protein expression profiles in NK92 cell lines at resting stage. Beta Actin and VDAC1 are used as cytosolic and mitochondrial protein as loading controls. n=3 independent experiments. Ordinary one-way ANOVA was used to compare groups.

Figure S2

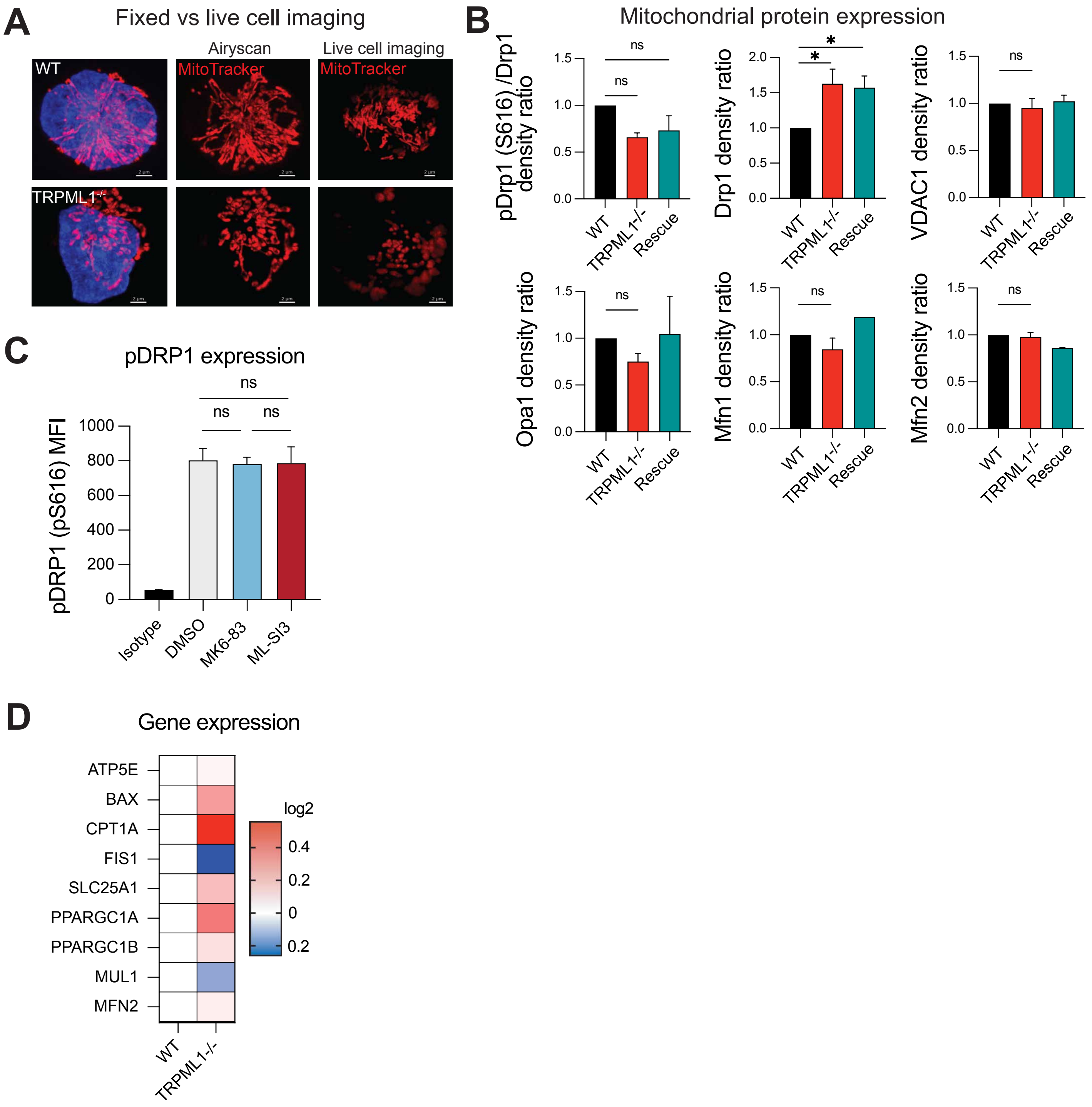

Supplementary Figure 2. (A) Side-by-side representation of maximum projection AiryScan or confocal images of 4% PFA-fixed (left panel) and live (right panel) NK92 cells depicting MitoTracker stained mitochondrial morphology in individual cells. Live cell imaging data was used as control for putative fixation-induced morphological artefacts. Hoechst (blue) was used as nuclear counterstain. Scale bar 2 μm. (B) Band intensity quantification for expression levels of mitochondrial fission proteins phosphorylated Drp1, total Drp1, and mitochondrial fusion proteins VDAC1, OPA1, Mfn1, Mfn2. Band intensities of NK92ML1-/- and NK92rescue cells is normalized to the intensity of NK92WT cells. n=3. Representative western blot image is shown as Supplementary Fig. S1C. (C) Flow cytometry-based quantification of mean fluorescence intensity of phosphorylated Drp1 expression in primary NK cells treated with TRPML1 activator MK6-83 [10 μM] or inhibitor ML-SI3 [10 μM] for 2h. Summary graph of n=3 independent experiments. (D) qPCR of key mitochondrial and metabolic genes. ATP synthase F1 subunit epsilon (ATP5E), Bcl-2-associated X protein (BAX), Carnitine palmitoyl transferase I (CAPT1), Mitochondrial fission 1 protein (FIS1), Mitochondrial tricarboxylate transport protein (SLC25A1), PPARG coactivator 1 alpha and beta (PPARGC1A, PPARGC1B), Mitochondrial E3 ubiquitin protein ligase 1 (MUL1), Mitofusin 2 (MFN2). Expression value of each gene is calculated relative to housekeeping gene Beta Actin or HPRT expression and presented as normalized to WT. n=3 independent experiments. Ordinary one-way ANOVA was performed for graphs (B-D).

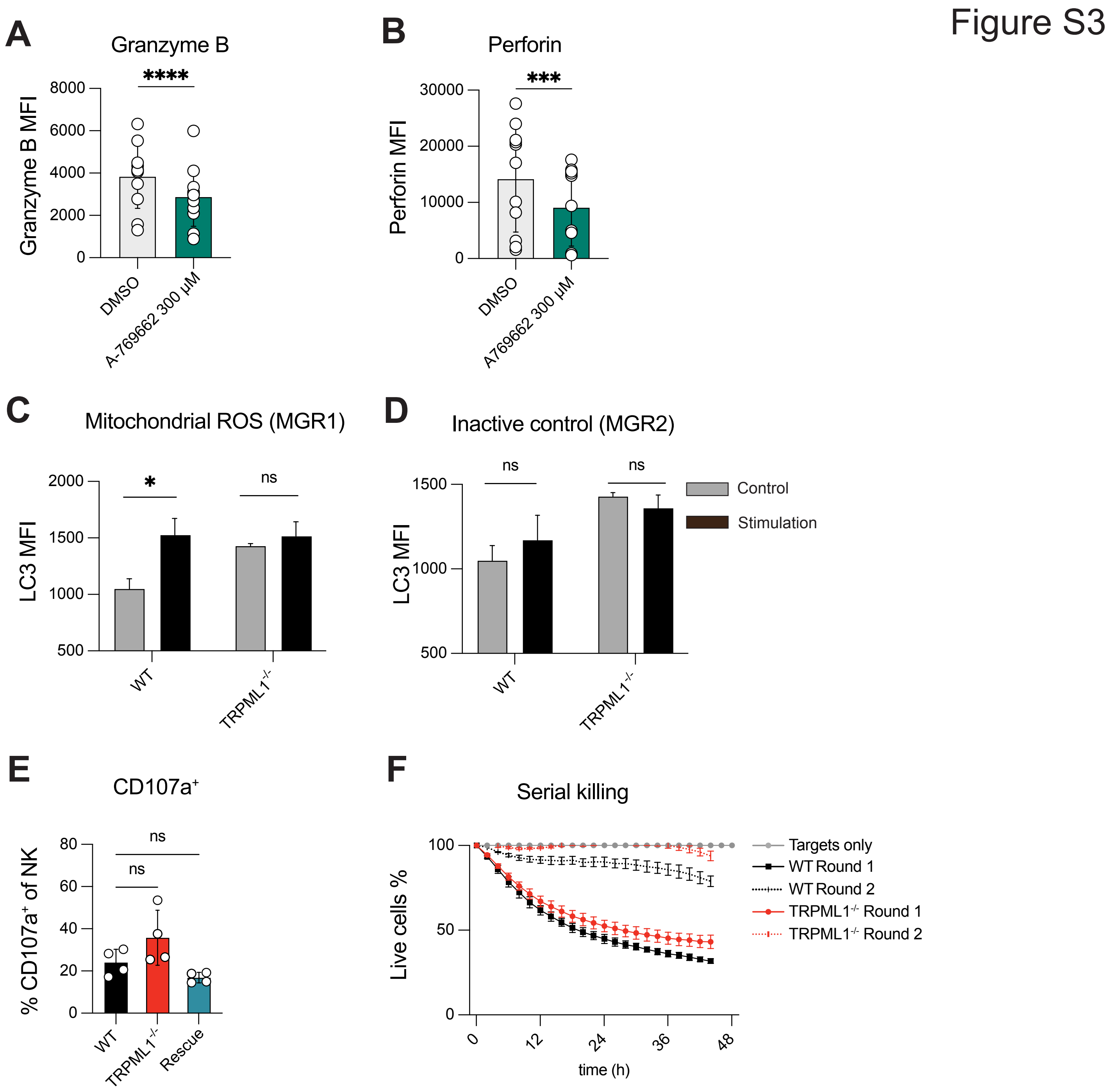

Supplementary Figure 3. (A) Granzyme B expression and (B) perforin expression in resting primary NK cells after overnight treatment with the AMPK agonist A769662 [300 µM]. Data is shown from 5 donors. (C) Membrane-bound LC3 levels after stimulation of NK92WT cells with vehicle control (grey bar) or a mitochondrial ROS-inducer MGR1 [1 µM]. 3 independent experiments were analyzed. (D) Membrane-bound LC3 levels after stimulation of NK92WT cells with vehicle control (grey bar) or MGR2 [1 µM] used as the inactive version of MGR1. 3 independent experiments were analyzed. (E) Degranulation response of NK92 cells against K562 tumor cells measured by surface mobilization of CD107a. 4h co-culture in the presence of anti-CD107a antibody. Summary graph of n=4 independent experiments. (F) Cytotoxicity assay of NK92 cells against K562 tumor cells at an effector to target ratio of 3:1 over 48h (round 1). At the end of 48h all effector cells were transferred into a new plate (round 2) with K562 target cells for another 48h. Representative graph shown for three independent experiments. Each dot represents the mean ±SEM of quadruplicates. Paired t - test was performed for (B-E) and Kruskal-Wallis with Dunn's multiple comparison t-test for (F).
